# Enhancing Robustness in UDC Image Restoration Through Adversarial Purification and Fine-Tuning

**DOI:** 10.3390/s25113386

**Published:** 2025-05-28

**Authors:** Wenjie Dong, Zhenbo Song, Zhenyuan Zhang, Xuanzheng Lin, Jianfeng Lu

**Affiliations:** 1 School of Computer Science and Engineering, Nanjing University of Science and Technology, Nanjing 210094, China; dongwj@njust.edu.cn (W.D.); songzb@njust.edu.cn (Z.S.); zhangzhenyuan@njust.edu.cn (Z.Z.); 2 School of AI and Advanced Computing, Xi’an Jiaotong-Liverpool University, Suzhou 215123, China; xuanzheng.lin23@student.xjtlu.edu.cn

**Keywords:** under-display-camera image restoration, deep learning, adversarial attacks, adversarial defense

## Abstract

This study presents a novel defense framework to fortify Under-Display Camera (UDC) image restoration models against adversarial attacks, a previously underexplored vulnerability in this domain. Our research initially conducts an in-depth robustness evaluation of deep-learning-based UDC image restoration models by employing several white-box and black-box attacking methods. Following the assessment, we propose a two-stage approach integrating diffusion-based adversarial purification and efficient fine-tuning, uniquely designed to eliminate perturbations while retaining restoration fidelity. For the first time, we systematically evaluate seven state-of-the-art UDC models (such as DISCNet, UFormer, etc.) under diverse attacks (PGD, C&W, etc.), revealing severe performance degradation (DISCNet’s PSNR drops from 35.24 to 15.16 under C&W attack). Our framework demonstrates significant improvements: after purification and fine-tuning, DISCNet’s PSNR rebounds to 32.17 under PGD attack (vs. 30.17 without defense), while UFormer achieves a 19.71 PSNR under LPIPS-guided attacks (vs. 17.38 baseline). The effectiveness of our proposed approach is validated through extensive experiments, showing marked improvements in resilience against various adversarial attacks.

## 1. Introduction

The advent of Under-Display Camera (UDC) sensors marks a significant leap in the evolution of display technologies, particularly in the realm of smartphones and other personal electronic devices. UDCs offer an innovative solution to the long-standing challenge of balancing screen real estate with camera placement by seamlessly integrating camera sensors beneath display screens. This technology enables a full-screen experience without notches or punch-hole cameras, thus revolutionizing modern devices’ design and aesthetic appeal. However, integrating cameras under display panels introduces complex challenges in image restoration, primarily due to the interference of the display pixels with the camera’s field of view. This results in inevitable image degradation issues like flare, blurring, color distortion, and contrast reduction [1].

To address these challenges, evolving deep-learning-based image restoration methods [1,2,3,4,5,6,7,8,9,10,11] have been proposed in recent years for the recovery and enhancement of degraded UDC images. Most of these methods focus on modeling the complex degradation process inherent in UDC systems and innovating new deep neural network architectures to enhance image restoration efficacy. However, the robustness of UDC image restoration models, particularly their resilience to subtle and often undetectable perturbations like adversarial attacks, has not been thoroughly explored. Given the prevalent application of UDC technology in smartphones, the vulnerability of UDC image restoration models to cyber threats, especially adversarial attacks, has emerged as a crucial area of concern. Such adversarial attacks can significantly aggravate existing image quality issues, posing a considerable risk to both the functionality and dependability of these devices. This underscores the need for research on UDC image restoration quality and the resilience and robustness of these deep models against adversarial attacks. This study is the first to address the vulnerability of UDC image restoration models towards adversarial attacks, highlighting the critical need for robust defense mechanisms in this domain.

**Figure 1:**
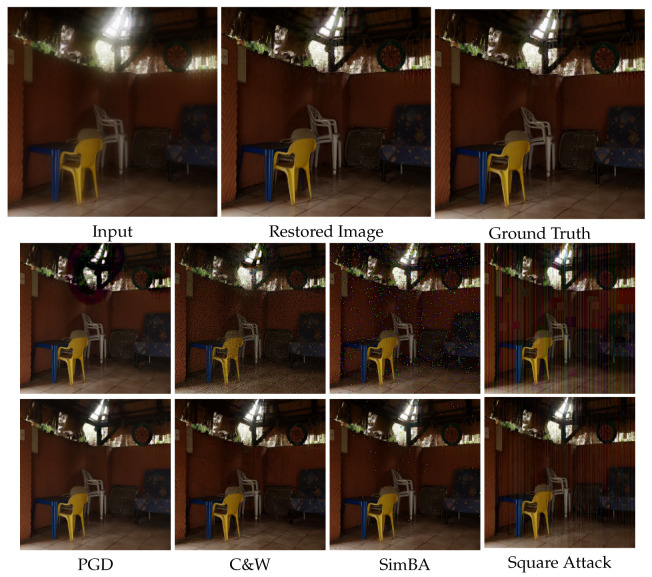
The visualization results of our proposed defense strategy under different attacks. The second and third rows represent the results without any defense and with our proposed defense strategy, respectively. Best view by enlarging the image.

In this paper, we first comprehensively evaluate the adversarial robustness of deep-learning-based UDC image restoration models. To make a fair comparison of different models, we concentrate on the blind UDC image restoration approaches, ignoring those using particular degradation priors, i.e., Point Spread Function (PSF) [12]. A total of 7 networks are considered in our research, including architectures of CNN [13], dynamic CNN [14], transformer [15,16,17,18], etc. We employ various sophisticated white-box and black-box attacking methods for a rigorous robustness evaluation process. White-box attacks have full knowledge and access to the models, making them ideal for thorough robustness testing. In contrast, black-box attacks have limited or no knowledge, thus close to real-world attack scenarios. Specifically, we use PGD (Project Gradient Descent) [19] and C&W (Carlini & Wagner) [20] for white-box attacks, and SimBA [21] and Square Attack [22] for black ones. This robustness evaluation is pivotal, as it reveals the weaknesses of the current UDC image restoration models and sets the stage for developing effective defense pipelines. Furthermore, we propose a novel defense framework that synergistically combines adversarial purification with fine-tuning processes to address adversarial vulnerabilities. Our approach leverages diffusion-based techniques [23,24], effectively negating the malicious perturbations introduced by adversarial attacks. Adversarial examples are purified before being fed into image restoration models. Subsequently, we employ fine-tuning methodologies to enhance the image restoration models further. This step is tailored to reinforce the models’ resilience against adversarial manipulations while improving their generalization to the original and purified images. Additionally, our fine-tuning strategy offers a more efficient pathway to achieving robust model performance than traditional adversarial training methods.In Figure 1, we can observe the effectiveness of our method.

Overall, the primary contributions of this paper are three-fold: (1) We conduct a comprehensive evaluation on the adversarial robustness of current UDC image restoration models, where a range of white-box and black-box attacking methods are employed, offering an in-depth understanding of models’ vulnerabilities and resilience against various adversarial threats. (2) We propose a novel defense strategy that combines state-of-the-art adversarial purification and fine-tuning techniques, providing an effective and efficient way to obtain a robust restoration model. (3) We carry out extensive experiments to investigate the robustness of the proposed defense method, which validates the achievement of more robust, reliable, and trustworthy UDC image restoration methods.

## 2. Related Work

### 2.1. UDC Image Restoration

To the best of our knowledge, Zhou et al. [1] were the first to address this new image restoration challenge using deep learning, along with their 2020 ECCV challenge [25]. They created the Monitor-Camera Imaging System (MCIS) to enable the collection of real paired data in a phone Pentile OLED (P-OLED) and a 4K Transparent OLED (T-OLED). They also used a model-based data synthesis pipeline to produce point spread functions (PSF) and UDC data from only display patterns and camera measurements. They introduced a UNet-based [26] architecture for image denoising and deconvolution. To obtain a larger receptive field and save memory, Sundar et al. [27] proposed a Deep Atrous Guided Filter network (DAGF), which utilizes restored low-resolution images to guide the restoration of high-resolution images. Feng et al. [3] considered the significant impact of high dynamic range on data generation and PSF measurement. They used rigid UDC components instead of manually covering the camera with regular OLED. Their proposed DynamIc Skip Connection Network (DISCNet) fully utilizes the conditional constraints of a long-tailed PSF to estimate the latent Clean image. Koh et al. [4] proposed a dual-branch network that handles high-frequency and low-frequency components separately. They introduced affine transformation connections to eliminate noise and preserve the structure of the image. Liu et al. [5] proposed a U-shape network to capture multiple spatial feature transformations. To balance the influence of pixels with different intensities in UDC images. Luo et al. [6] identified unique statistical characteristics of UDC and ground-truth images in HSV space. They proposed a cascaded curve estimation network to enhance UDC images by adaptively fitting the estimated images in H and S channels. Zhou et al. [7] introduced GAN [28] to generate paired UDC images and proposed a transformer-based network to restore images. Song et al. [8] considered the real physical scattering effects and utilized them to guide the image branch to restore an explicit scene. Current research mainly relies on synthetic data and complex network architectures, which have improved image restoration performance but face limitations in real-world generalization and computational resource-intensive bottlenecks. Meanwhile, issues such as simplified physical models, insufficient real-time performance, and privacy risks have constrained its practical application and large-scale deployment in mobile UDC systems.

### 2.2. General Adversarial Attacks and Defenses

Deep neural networks (DNNs) can misclassify images under the influence of imperceptible perturbations [29,30]. Based on the adversary’s understanding of the victim model, existing adversarial attacks can be divided into white-box attacks (where all the information about the target model is known) and black-box attacks (where only the output results of the target model for input samples can be observed). The former mainly uses the gradient information of the network to generate adversarial samples, e.g., L-BFGS [29], FGSM [31], DeepFool [32], PGD [19], and C&W [20]. The latter mainly uses input-output model queries to generate adversarial samples, e.g., ZOO [33], SimBA [21], GeoDA [34], and Square Attack [22]. Attackers not only target computer vision tasks [35,36,37], but also try to harm other DNN-based tasks, such as natural language processing [38] and image generation [39].

To protect DNNs from adversarial attacks, adversarial defenses have evolved into two main branches: adversarial training and adversarial preprocessing. Adversarial training [29,40] involves training the network with adversarial samples, which enhances its robustness. However, this technique can make the network become overly specialized in countering a specific type of attack, thereby diminishing its performance on clean images [41,42]. Adversarial purification [43,44] is one of the typical preprocessing methods, aiming to transform adversarial samples into clean samples. Generative methods such as GAN [28], VAE [45], and Diffusion [23] has been widely applied, for example, Defense-GAN [46], A-VAE [47], and DiffPure [24].

### 2.3. Adversarially Robust Image Restoration

Recent research has explored the topic of adversarial attacks in low-level image restoration tasks, such as derain [36], super-resolution [48,49,50], dehaze [37], deblur [35], and reflection removal [51]. Yu et al. [36] systematically investigated the impact of critical modules on the robustness of rain removal models against adversarial attacks. They evaluated the models’ ability to withstand adversarial attacks from both human and machine vision perspectives and the impact on downstream tasks. Yue et al. [50] focused on eliminating adversarial noise in the frequency domain of super-resolution models. Gui et al. [37] proposed five adapted attack methods for the dehaze problem. Song et al. [51] conducted a comprehensive study on the robustness of reflection removal models against adversarial attacks, considering different attack methods, attack levels, and attack regions. They proposed a robust reflection removal model integrating cross-scale attention modules, multi-scale fusion modules, and an adversarial image discriminator.

However, there is currently a lack of research on robust networks designed to defend against adversarial attacks in UDC image restoration. It is necessary to evaluate the impact of these attacks and to develop appropriate attack methods for a comprehensive assessment.

In this work, we explore the impact of adversarial attacks on the robustness of image restoration. We have chosen white-box and black-box attacks to comprehensively examine the robustness of UDC recovery methods under adversarial attacks.

## 3. Adversarial Attacks on UDC IR

Similar to adversarial attacks on other image restoration tasks [35,51], the critical element in UDC adversarial attacks is the creation of visually imperceptible perturbations on the input images. The perturbations must be specifically tailored to the restoration models, ensuring they do not substantially degrade the visual quality. This aspect is vital, as the primary objective of adversarial attacks is to subtly influence the outcomes of the restoration process through the introduction of noise rather than to overtly reduce the quality of UDC images, which would merely increase the complexity of the restoration task. We incorporate sample-specific noise maps into the input UDC images pixel-wise while limiting the range of perturbations to ensure they are visually indistinguishable.

Mathematically, given x as a UDC image, the adversarial attack generates a pixel-wise noise map δ according to the image restoration model f(·;ψ), where ψ represents the parameters of the model. The goal is to create an adversarial example $x_{adv}$ from an original image x, such that the perturbation δ is small but effective enough to fool the model. This can be formulated as:

(1) $$x_{adv}=x+\delta ,\;\mathrm{where}\;{∥\delta ∥}_{\ell }<\epsilon$$

Here, ϵ is a small constant that controls the magnitude of the perturbation. Given the original restored image y without adversarial attacks, where y=f(x;ψ), the adversarial example is crafted to maximize the loss function $J(f\left(x_{adv};\psi \right),y)$:

(2) $$\delta =\arg\max_{{∥\delta ∥}_{\ell }<\epsilon }J\left(f\left(x_{adv};\psi \right),y\right)$$

The loss function J(·,·) quantifies the disparity between restored images with and without adversarial attacks. Accordingly, there are two types of loss functions utilized in adversarial attacks [51]. The first type centers around pixel-wise image discrepancy, commonly measured by metrics like Mean Squared Error (MSE). Conversely, the second objective prioritizes the high-level perceptual similarity of output images, such as LPIPS [52]. The lower the value of LPIPS(Learned Perceptual Image Patch Similarity), the more similar the two images are, and vice versa, the greater the difference.

### 3.1. White-Box Attacks

For white-box attacks, the attackers have full access to the UDC image restoration model. This is, in Equation (2), the architecture of *f* and the parameters ψ are known to the attackers. Thus, we choose two typical adversarial attackers, the PGD and the C&W.

PGD is an iterative method commonly used for adversarial training and robustness testing of neural networks [35,51]. It works by making small, calculated adjustments to the input UDC image x in the direction that increases the loss function $J(f\left(x_{adv};\psi \right),y)$, while ensuring that $∥x_{adv}{-x∥}_{\ell }<\epsilon$. Here, *ℓ* indicates the chosen norm, typically *L*-*∞*, that bounds the adversarial perturbations.

Compared to PGD, C&W focuses on minimizing the perturbations δ, resulting in more subtle and harder-to-detect adversarial examples. This method is mainly known for its precision and effectiveness in crafting less perceptible perturbations. Especially in its L2 variant, it aims at creating the smallest possible perturbations.

### 3.2. Black-Box Attacks

For black-box attacks, the attackers can only observe the outputs of UDC image restoration models. In other words, there is only information about the clean outputs y, as well as the adversarial outputs $f(x_{adv};\psi )$. Hence, δ is generated using a substitute restoration model or trial-and-error methods. This paper applies the SimBA and Square attack approaches to perform black-box attacks.

SimBA [21] operates by randomly perturbing individual pixels or small groups of pixels in the UDC image and observing the changes in the model’s output. SimBA does not require gradient information or internal knowledge of the restoration model, making it broadly applicable and straightforward to implement.

Square Attack [22] is another black-box method that generates adversarial examples by modifying a random contiguous square area in the UDC image. This approach is more query-efficient than SimBA, often achieving higher success rates with fewer queries. However, this might result in more visually noticeable perturbations compared to pixel-wise methods like SimBA.

## 4. Adversarial Purification and Fine-Tuning

Existing image restoration models accomplish adversarial robustness by predominantly analyzing the contributions of network modules to robustness. With a combination of robust modules, novel network architectures are designed to detect or remove adversarial perturbations. Moreover, adversarial training techniques are incorporated to bolster the model’s resilience further. However, adversarial training can be time-consuming and inefficient. Furthermore, training for robustness often necessitates a performance trade-off between clean (unperturbed) images and adversarial (perturbed) images, which can adversely impact the restoration quality for clean inputs [53].

**Figure 2:**
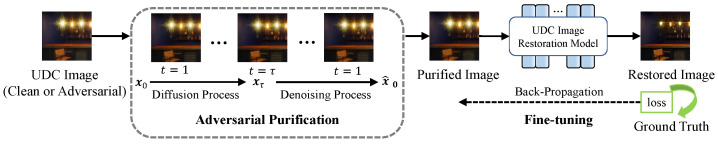
The overall pipeline of our proposed defense strategy for under-display camera image restoration.

Instead of constructing new neural network models, we propose to purify adversarial perturbations. Specifically, we utilize a preprocessing module to remove adversarial noise from input UDC images. The processed UDC images can still be used for the original network or module. Ideally, any pre-trained UDC image restoration models can be seamlessly integrated after the purification. However, the purification could also denoise the degradation of UDC images to a certain degree, causing domain discrepancy to the original UDC images. To accelerate the adaptation of the restoration model to the purified outputs, we create a specialized fine-tuning strategy without the need for adversarial training data. The fine-tuning facilitates more effective and rapid model adjustment to the purified data. The whole defense pipeline is illustrated in Figure 2.

### 4.1. Adversarial Purification

Adversarial purification aims to neutralize the impact of adversarial perturbations in the input UDC images. Let ${\hat{x}}_{0}$ be the purified image, and the purification process is modeled as $p_{\phi }\left({\hat{x}}_{0}|x\right)$. Here, ϕ denotes the parameters of the purification model. Inspired by [24,54,55], x is first corrupted to a noisy status through a diffusion process, typically using DDPM [23]. DDPM(Denoising Diffusion Probabilistic Models) is a diffusion model. For simplicity, we denote the x as $x_{0}$ at timestep 0, and the forward process is formulated as:

(3) $$q\left(x_{t}|x_{t-1}\right)=N\left(x_{t};\sqrt{1-\beta_{t}}x_{t-1},\beta_{t}I\right)$$

where $x_{t}$ is the data at timestep *t*, $\beta_{t}$ is the pre-defined noise level, and N denotes the normal distribution. Based on the Markov Chain, for a specific timestep τ, the noisy distribution can be calculated as:

(4) $$\begin{array}{cc} q(x_{\tau }|x_{0}) & =N(x_{\tau };\sqrt{{\bar{\alpha }}_{\tau }}x_{0},\sqrt{1-{\bar{\alpha }}_{\tau }}I)\\ \; & =\sqrt{{\bar{\alpha }}_{\tau }}x_{0}+\sqrt{1-{\bar{\alpha }}_{\tau }}\epsilon \end{array}$$

Here, ${\bar{\alpha }}_{\tau }$ represents the cumulative multiplication from t=1 to t=τ, and ϵ follows the standard normal distribution N(0,I). As τ increasing, the distribution of $x_{\tau }$ eventually satisfies N(0,I). Intuitively, the adversarial image $x_{adv}$ could also be diffused as $x_{adv}^{\tau }∼N\left(0,I\right)$ when the timestep τ is large enough. Thus, the clean and adversarial image distributions get closer over the forward diffusion process [24]. This indicates that the adversarial perturbations could be gradually purified by adding specific noises. Since adversarial perturbations are typically small, there is no need for many timesteps to disperse the input UDC image. Empirically, around 100 steps are sufficient to attain close enough noisy images, whether inputting the adversarial image or the corresponding clean one.

Subsequently, the reverse process in DDPM is used to reconstruct the purified image ${\hat{x}}_{0}$ from the noisy image. It can be described as a reverse Markov Chain that iteratively denoises the data at each timestep. The reverse process for a specific timestep *t* is given by:

(5) $$p_{\theta }\left(x_{t-1}|x_{t}\right)=N\left(x_{t-1};\mu_{\theta }\left(x_{t},t\right),\Sigma_{\theta }\left(x_{t},t\right)\right)$$

Here, $\mu_{\theta }\left(x_{t},t\right)$ and $\Sigma_{\theta }\left(x_{t},t\right)$ are the mean and covariance of the Gaussian distribution at timestep *t*, which are parameterized by the neural network with parameters θ. Starting from $x_{\tau }$, the reverse process iteratively refines $x_{t}$ to approximate ${\hat{x}}_{0}$, and finally accomplishes the purification pipeline.

Since the DDPM is typically trained with high-quality images, the purified image may not maintain the UDC features. For some image restoration tasks, e.g., face restoration [55], the adversarial purification could even become a degradation remover. This forms new challenges for pre-trained UDC image restoration networks because these networks are trained specially for such degradation patterns. They have less generalization to recover the purified UDC image. Hence, the fine-tuning strategy is further proposed and introduced as follows.

### 4.2. Model Fine-Tuning

To fine-tune the UDC image restoration model, we generate purified images with different timesteps for increasing sample diversity. As mentioned above, purified images should share the same distribution for clean and adversarial UDC inputs. Hence, adversarial training is not necessary for the model fine-tuning. Moreover, during the fine-tuning phase, the parameters of the purification network are held constant, ensuring stability and consistency in the purification process.

For symbolic expression, let g(·,t;ϕ) denote the purification model. The purified UDC image $x_{pur}$ is obtained by $x_{pur}=g\left(x,t^{*};\phi \right)$. Unlike the real purification phase, there is no need for a strictly large enough timestep $t^{*}$. Limited diffusion and denoising steps could also help improve the diversity of UDC samples. Through this method, training efficiency can be greatly raised.

Regarding the loss function for fine-tuning, we employ supervised losses to minimize the discrepancy between the new prediction and the ground truth. Let L(·,·) represent the in total loss function. The fine-tuning can be formulated as:

(6) $$\psi^{*}=\arg\min_{\psi }L\left(f\left(x_{pur};\psi \right),x_{gt}\right)$$

where $x_{gt}$ the ground truth image without UDC effects. The loss function is a linear combination of typical image reconstruction loss terms, such as Euclidean loss, GAN loss, perceptual loss, etc. We adaptively utilize loss terms consistent with training the original UDC image restoration models.

Our approach mitigates the immediate effects of adversarial attacks through these two stages and reinforces the model’s long-term resilience to such perturbations.

The above scheme partially utilizes existing methods, but the overall combination was first proposed in this paper, and our efficiency is higher.

## 5. Experiments

### 5.1. Implementation Details

**Dataset.** For the dataset, following [3,8], we synthesize the dataset using nine existing ZTE Axon 20 phone (ZTE, Shenzhen, China), PSFs and one real-scene PSF [56]. We generate a total of 21,060 training image pairs and 3600 testing image pairs from the 2016 training images and 360 testing images, which are collected from the HDRI Haven dataset [57].
**Experimental details.** Specifically, we adopt a supervised loss function that combines L2 reconstruction loss and perceptual loss based on VGG-19 features (relu3_3 and relu4_2). For models involving adversarial training, the adversarial loss is also incorporated. The learning rate is initialized at $2\;\times \;10^{-4}$ and decayed using cosine annealing to $1\;\times \;10^{-6}$. Fine-tuning is conducted with a batch size of 16. The training set is derived from the same synthetic dataset, using purified images generated via the DDPM process with varying diffusion steps (e.g., 50–100) to improve data diversity. Data augmentation includes random horizontal flips, 256 × 256 cropping, and light color jittering. For white-box attacks, PGD uses 20 iterations with step sizes of 1/256, 2/256, 4/256, and 8/256 under different difficulty levels, while C&W is implemented using its L2 version with 9 binary search steps and zero confidence. For black-box attacks, SimBA is conducted with 1000 iterations and a step size of 4/256. All experiments are performed on a server equipped with 8 NVIDIA RTX 3090 GPUs (Nvidia, Santa Clara, CA, USA) and 2 Intel Xeon Silver 4314 CPUs (Intel, Santa Clara, CA, USA). Runtime measurements are obtained using standard Python 3.8 timing tools and reported as the average inference time over all test images.
**UDC IR methods.** We select six state-of-the-art UDCIR (Under-Display Camera image restoration) methods for comparison, including DAGF (Deep Atrous Guided Filter) [27], DISCNet (Dynamic Skip Connection Network) [3], UDCUNet (Under-Display Camera Image Restoration via U-shape Dynamic Network) [5], BNUDC (A Two-Branched Deep Neural Network for Restoring Images from Under-Display Cameras) [4], SRUDC (Under-Display Camera Image Restoration with Scattering Effect) [8], and DWFormer (Dynamic Window Transformer) [7]. We also include a general image restoration method called UFormer (U-Shaped Transformer) [58] for a comprehensive evaluation. All the methods are re-trained on the training dataset with the same parameter set to ensure a fair comparison. We adopt PSNR (Peak Signal-to-Noise Ratio) [59] and SSIM (Structure Similarity Index Measure) [52] as the evaluation metrics.

### 5.2. Robustness Evaluation on Attack Methods Comparison

**From the aspect of PSNR.** Table 1 compares the PSNR performance of various deep UDCIR methods under different attack methods. All methods experience a significant decrease in performance after being subjected to adversarial attacks. DISCNet demonstrates the best performance in clean images with the PSNR of 35.237. UDCUNet and DAGF also perform well, with the PSNR of 27.427 and 24.911, respectively. DWFormer and UFormer exhibit relatively lower performance. This can be attributed to the fact that DWFormer was trained on the TOLED&POLED dataset [1] and has a more significant number of parameters. The limited size of the training set and the larger model size prevent UFormer from fully leveraging its performance capabilities.

To comprehensively analyze the impact of different attacks on the robustness of various methods, we present our findings from two perspectives:

1. **Single attack method on different restoration methods.** In terms of PGD, this type of attack generally decreased the performance of all models. The performance of DAGF witnessed the most substantial decline, dropping from 24.972 to 13.772. BNUDC experienced the second most prominent decrease, going from 24.911 to 14.565. However, DISCNet and UDCUNet showed better restoration performance, indicating their robustness in handling such attacks. In terms of C&W, most models saw a significant decline in performance, particularly DISCNet and UDCUNet. UFormer, on the other hand, demonstrated significant robustness. In terms of SimBA, the impact of attacking UDCIR methods was relatively minor, with no significant vulnerabilities exposed. DISCNet and BNUDC exhibited a slight advantage in maintaining stable performance. In terms of square attack, the results showed notable differences in impact across different models. UFormer and DISCNet were more effective in dealing with such attacks, while DWFormer was particularly sensitive to square attacks, exhibiting a considerable decline in performance.
2. **Single restoration method against different attack methods.** DISCNet exhibits overall strong performance, excelling in handling clean images and maintaining high effectiveness under various attack types. However, it shows notable sensitivity to C&W attack, suggesting a potential weakness in its resilience against this specific type of adversarial attack. UDCUNet demonstrates robustness across attack scenarios, showcasing balanced performance under various adversarial conditions. Despite this, the model reveals a sensitivity to C&W attack, with a noticeable decline in performance when subjected to this particular form of attack. BNUDC stands out for its excellent performance under SimBA attacks, showcasing superior capabilities in the face of this specific adversarial technique. However, its performance experiences significant drops under other attack types, mainly exhibiting vulnerability to C&W attack, indicating the need for additional optimization or protective measures. DWFormer reveals vulnerability to square attack, displaying the weakest performance under this specific adversarial condition. Its general performance is average across other attack types and clean image processing, lacking standout achievements compared to its counterparts. UFormer demonstrates remarkable resilience under C&W attacks, highlighting its strength in maintaining robustness against this particular form of adversarial assault. The model exhibits balanced performance, showing competency in handling clean images and various attack types without specific weaknesses.

In summary, the results of the robustness evaluation reveal that the current UDCIR methods are vulnerable to adversarial attacks. The performance of these models is significantly degraded under various adversarial conditions, particularly when subjected to C&W attack. DISCNet demonstrates superior performance when dealing with clean images, boasting the highest clean accuracy. However, in terms of robustness, UFormer exhibits relatively better performance across various attack methods, including PGD, C&W, SimBA, and SquareA, showcasing a more comprehensive resilience.

**From the aspect of SSIM.**Table 2 compares the SSIM performance of these methods under different attack methods. Overall, DISCNet stands out as the most effective method. It leads with 0.960 in processing clean images and maintains the top position with an average performance SSIM of 0.551 under four different attack methods. This indicates that DISCNet is best suited to deal with unattacked clean images and demonstrates the strongest resilience and best overall performance under various attacks. To obtain a more detailed and comprehensive understanding of the robustness of different UDCIR methods against various attack methods, we analyze this table from two perspectives:

1. **Single attack method on different restoration methods.** Regarding specific attack methods, different methods have their respective strengths. For instance, while DISCNet shows the best resistance against PGD and Square attack, BNUDC and SRUDC perform better under SimBA. UFormer, on the other hand, indicates relatively better performance against the C&W attack.
2. **Single restoration method against different attack methods.** In terms of PGD attack, DISCNet demonstrates superior resilience, achieving the highest SSIM of 0.907. This indicates its effectiveness in countering this type of attack. Conversely, UDCUNet and UFormer show more vulnerability with SSIM of 0.660 and 0.543, respectively, suggesting a greater sensitivity to PGD attacks. In terms of the C&W attack, a notable shift in performance is observed. UFormer emerges as the most resistant, with the SSIM of 0.441, indicating its strength against this sophisticated attack method. Conversely, SRUDC shows significant vulnerability, scoring only 0.019, highlighting its weak defense against CW attacks. In terms of SimBA, BNUDC, and SRUDC exhibit commendable resilience, scoring 0.805 and 0.715, respectively. These scores reflect their robustness in scenarios where the attacker’s strategy is not fully known, a key strength for black-box attack scenarios. In contrast, DISCNet and DAGF show comparatively weaker performance under SimBA attacks. In terms of Square Attack, DISCNet again stands out, with the SSIM of 0.559, which underscores its effectiveness in repelling this efficient and effective attack. Conversely, DWFormer demonstrates a significant lack of resistance with a score of 0.019, pointing to its vulnerability to Square Attack.

In conclusion, the robustness evaluation indicates that current UDCIR methods are susceptible to adversarial attacks. DISCNet [3] demonstrates excellent performance when handling clean images. However, in terms of robustness, UFormer [58] exhibits relatively better performance across various attack methods.

### 5.3. Robustness Evaluation on Attack Objectives Comparison

**Comparison of different attack objectives in PGD in terms of PSNR.** Table 3 shows the PSNR performance of various deep UDCIR methods under different attack objectives in the PGD attack. Analyzing the data reveals a trend indicating that the MSE objective tends to induce more pronounced degradation than the LPIPS objective. For DISCNet, SRUDC, DWFormer, DAGF, and UFormer, there is a marginal difference in performance following both types of attacks, with variations of approximately 1.000. However, BDUDC exhibits a notable contrast, experiencing a significant drop in PSNR from 24.911 to 14.565 under the MSE objective, while the LPIPS objective results in a PSNR decrease to 22.356 from 24.911. Although the MSE objective performs comparably to the LPIPS objective in more challenging restoration methods, it outperforms the latter in specific instances.
**Comparison of different attack objectives in PGD in terms of SSIM.** Table 4 compares the SSIM performance of different attack objectives in PGD. It can be observed that the MSE objective yields more effective results compared to the LPIPS objective for most restoration methods. DISCNet and BNUDC exhibit poorer robustness when facing the LPIPS objective, primarily attributed to their utilization of VGG-based [60] visual loss methods. Other methods are more susceptible to the MSE objective.

### 5.4. Robustness Evaluation on Attack Levels Comparison

**Figure 3:**
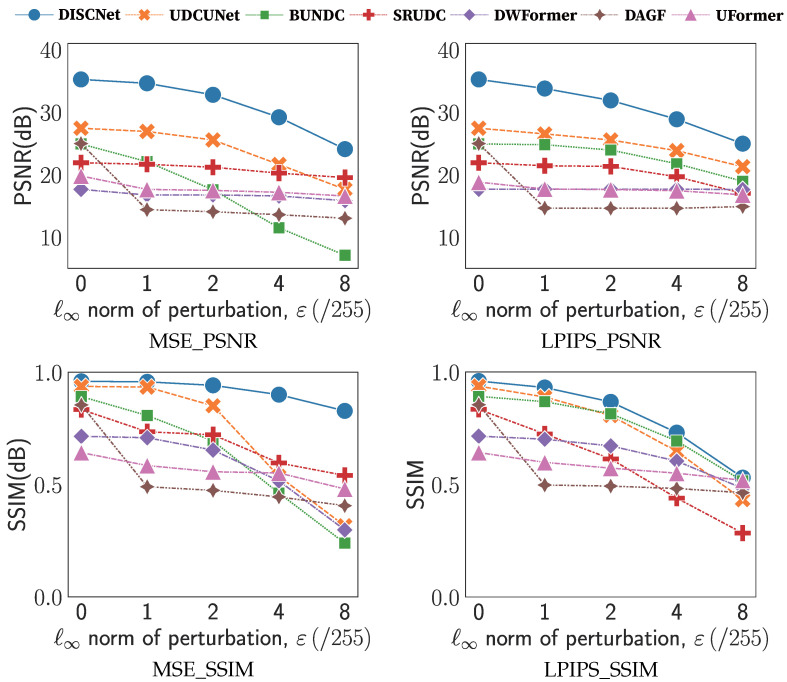
Comparison of the PSNR and SSIM values with respect to perturbation levels ϵ. ‘MSE’ and ‘LPIPS’ represent the MSE objective and LPIPS objective, respectively.

Figure 3 illustrates the variations of PSNR and SSIM under different perturbation levels. As the perturbation level increases, the output quality of all methods noticeably deteriorates. As shown in Figure 3, BNUDC exhibits the highest sensitivity to attack difficulty, with its restoration performance declining the most with increasing perturbation. DISCNet and UDCUNet also experience significant decreases. On the other hand, DWFormer, DAGF, and UFormer exhibit sudden drops after being subjected to minimal difficulty attacks, especially DAGF, but subsequently become less sensitive to noise. SRUDC maintains robustness throughout. In Figure 3, most methods show relatively moderate decreases. Only DAGF experiences a sudden drop when facing attacks, but remains insensitive afterward.

In terms of the SSIM, as shown in Figure 3, BNUDC and SRUDC demonstrate noticeable downward trends, indicating that the attack methods have a more significant impact on the structural aspects of the recovered images. DISCNet performs better under the MSE attack than the LPIPS (Learned Perceptual Image Patch Similarity) attack objective. The decline in UDCUNet is no longer gradual, as it experiences a significant drop when perturbation ϵ=4. Additionally, DWFormer and UFormer exhibit significant performance fluctuations after the attack, suggesting that their models are more sensitive to noise in restoring image structures.

### 5.5. Defense Strategy Results

As mentioned in Section 5.2 and Section 5.4, among all the methods evaluated, DISCNet [3] demonstrates superior restoration performance, while UFormer [58] exhibits exceptional robustness. To ensure a fair comparison, we conducted adversarial training on both methods. We extensively compared the results of adversarial training with our proposed defense strategy.

**PGD Objective Comparison.** Table 5 presents the PSNR performance of adversarial training and our proposed defense strategy with PGD attack. After undergoing adversarial training, DISCNet exhibits improved robustness against PGD attack, but its restoration performance on clean images slightly decreases from 35.237 to 34.082. UFormer, on the other hand, shows a significant improvement in robustness after adversarial training, while its performance on clean images remains relatively unchanged. When only implementing the DiffPure (DP) strategy, DISCNet experiences a more noticeable decline in performance on clean images, while UFormer shows a slight improvement. The DP strategy refers to the diffusion model of adversarial purification that first interferes with adversarial examples with noise through a forward diffusion process, and then reconstructs clean images through a reverse generation process to defend against attacks. This suggests that DISCNet is more sensitive to noise. However, when implementing our proposed defense strategy, UFormer demonstrates a significant improvement in performance on clean images compared to the original model, increasing from 18.795 to 19.312. DISCNet’s performance remains similar to the original model on clean images, but its robustness significantly improves when under attack.

Table 5 compares the SSIM performance of adversarial training and our proposed defense strategy in terms of different attack objectives in PGD. Upon the application of adversarial training, DISCNet manifests a nominal decrement in its efficacy on clean images, evidenced by a reduction in metric from 0.960 to 0.956. Concurrently, its robustness is an incremental enhancement against MSE attack and LPIPS attack adversarial vectors, with performance indices improving to 0.908 and 0.763, respectively. Integrating the DiffPure (diffusion models for adversarial purification) strategy into DISCNet precipitates a substantial diminution in its performance with clean images, descending from a score of 0.960 to 0.872. Nonetheless, this approach markedly bolsters its resilience against MSE attack and LPIPS attack, with performance indices ascending to 0.856 in both cases. The amalgamation of our proposed defense strategy in DISCNet results in a slight regression on clean images, decreasing from 0.960 to 0.945. However, this strategy culminates in a substantial elevation in its adversarial defense indices against MSE attack and LPIPS attack, soaring to 0.928 and 0.929, respectively.

In summary, our proposed defense strategy effectively elevates their robustness against adversarial attacks while concurrently preserving or augmenting their innate capabilities on clean images. It offers a balanced and productive solution to the clean challenge of bolstering adversarial robustness without eroding performance on clean images.

### 5.6. Other Attack Methods Comparison

**Figure 4:**
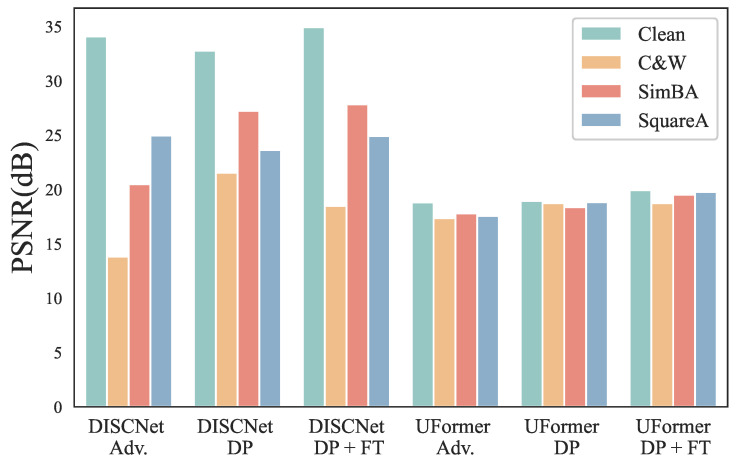
Comparison of adversarial training and our proposed defense strategy in terms of different attack methods on the synthetic dataset. DP refers to the utilization of DiffPure, whereas FT indicates the implementation of Fine-tuning.

In Figure 4, we also present the PSNR performance of adversarial training and our proposed defense strategy when facing C&W, square attack, and SimBA. Even after adversarial training, we can observe that DISCNet and UFormer still exhibit significant degradation under C&W attack, but they demonstrate better robustness against square attack. When only implementing the DP strategy, DISCNet and UFormer show noticeable improvements in robustness against C&W attacks, indicating that the DP strategy is more effective in defending against C&W attacks. However, in this case, we can see that both methods experience a decline in performance on clean images, attributed to DP’s introduction of additional small-scale noise. When implementing our proposed defense strategy, DISCNet, and UFormer also show significant improvements in robustness against these three attacks. At the same time, their performance on clean images is even better and surpasses that of the original model.

**Figure 5:**
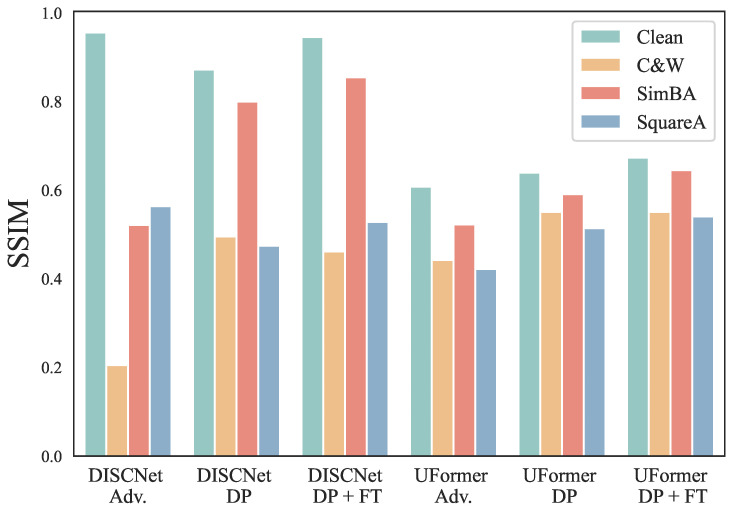
Comparison of SSIM for adversarial training and our proposed defense strategy in terms of different attack methods on the synthetic dataset. DP refers to the utilization of DiffPure, whereas FT indicates the implementation of Fine-tuning.

Figure 5 compares the SSIM performance of adversarial training and our proposed defense strategy in terms of other attack methods, e.g., C&W, SimBA, and square attack. DISCNet and UFormer exhibit significant degradation under the C&W attack, while both perform well in defending against SimBA. When only DP is implemented, DISCNet and UFormer show improved robustness against C&W and SimBA. After implementing our proposed defense strategy, DISCNet demonstrates the best robustness against SimBA and square attack, comparable to adversarial training under C&W attack, while maintaining excellent performance on clean images. UFormer showcases superior robustness across all attack methods while delivering outstanding performance on clean images.

### 5.7. Visual Comparison

**Figure 6:**
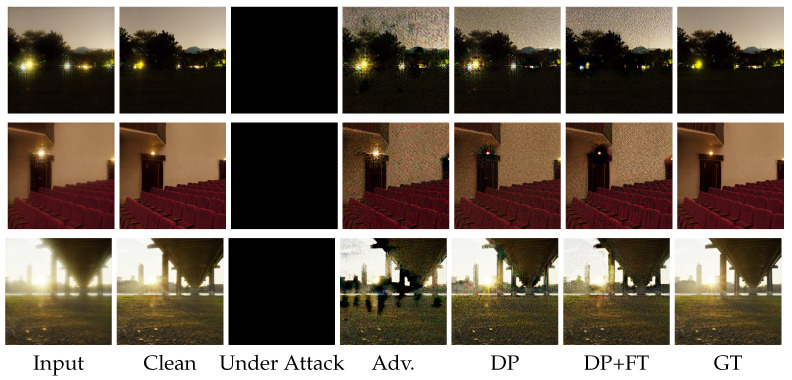
Comparison of DISCNet with adversarial training and our proposed defense strategy under C&W attack on the synthetic dataset. Best view by enlarging the image.

**Figure 7:**
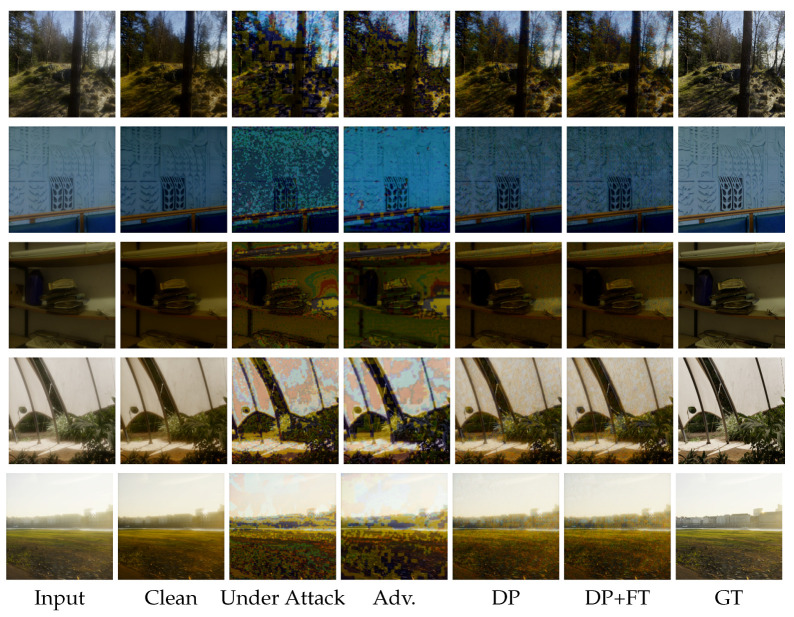
Comparison of UFormer with adversarial training and our proposed defense strategy under C&W attack on the synthetic dataset. Best view by zooming in.

**Figure 8:**
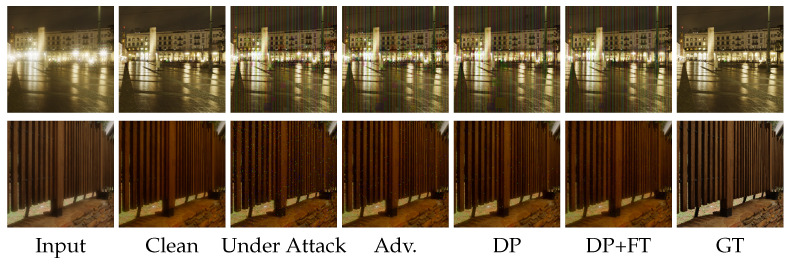
Comparison of adversarial training and our proposed defense strategy regarding different black attack methods on the synthetic dataset. The first row represents the performance of DISCNet [3] under Square attack. The second row represents the performance of UFormer [58] under SimBA, respectively. Best view by zooming in.

**Comparison of DISCNet under C&W attack with different defense strategies.** In Figure 6, we present visual results of DISCNet with adversarial training and our proposed defense strategy under C&W attack. DISCNet fails to perform image restoration after being attacked, resulting in a completely black image. When adversarial training is applied, the attacked images exhibit black color patches and noticeable glare. Light diffusion is a significant phenomenon in the second row of images. When only DP is applied, there is a noticeable improvement in glare and irregular color patches in the images. However, some irregular color patches are still present, especially in the second row of images. When our proposed defense strategy is implemented, the diffusion around the light source in the images of the second row is significantly reduced. Similarly, no irregular color patches are observed in the images of the third row.
**Comparison of UFormer under C&W attack with different defense strategies.** In Figure 7, we present additional visual results of the UFormer under the C&W attack. From the third column, it can be observed that UFormer exhibits numerous irregular patches when subjected to adversarial attacks, making it challenging to recover the original image. When adversarial training is applied, there is a slight improvement in the appearance of irregular patches in the visual results compared to the previous case. However, in the fifth and sixth columns, significant improvements in the appearance of irregular patches are observed when using both our proposed defense strategy and the only DP. Compared to the results obtained from clean images, our proposed defense strategy can preserve the overall structure of the original image and maintain good robustness.
**Comparison of DISCNet and UFormer under black attack with different defense strategies.**Figure 8 visually compares adversarial training and our proposed defense strategy when facing a square attack and SimBA. Based on the first-row images, it can be seen that DISCNet exhibits noticeably large and small blocks of colors when subjected to square attacks. When our proposed method is implemented, these blocks of colors are significantly reduced, and the glare issue is well resolved. Moreover, in the second-row images, it can be seen that UFormer performs better when subjected to Simba attacks with the application of our method.

## 6. Conclusions

In this research, we thoroughly investigated the robustness of Under-Display Camera (UDC) image restoration models against adversarial attacks. Our comprehensive evaluation, utilizing white-box and black-box methods, identified significant vulnerabilities in current deep-learning-based UDC image restoration models. We introduced a novel defense framework that synergizes adversarial purification with fine-tuning processes to address these challenges. The diffusion-based purification stage proved highly effective in mitigating adversarial perturbations. Subsequent fine-tuning further reinforced the models’ resilience, enhancing the quality and integrity of the restored images. Extensive experiments demonstrated considerable improvements in robustness against diverse adversarial attack types. This study contributes significantly to UDC image processing technology and offers insights for developing robust deep learning models for image restoration amidst evolving adversarial threats. The adversarial purification and fine-tuning framework proposed in this article significantly improves the robustness of the UDC image restoration model, but still has limitations: the multi-step iteration of the diffusion model leads to low computational efficiency and is difficult to meet real-time requirements; the defense effectiveness decreases in extreme disturbances or dynamic attack scenarios; the generalization ability of synthetic data validation is limited in real complex scenes such as dynamic lighting. Future research can be improved in the following directions: designing dynamic detection modules to achieve adaptive defense, using self-supervised learning to reduce dependence on paired data, and integrating multimodal sensor data to enhance system robustness. These improvements will promote the practicality and security of UDC technology in open environments, providing more reliable image restoration guarantees for smart devices.

## Figures and Tables

**Table 1:** Comparison of different attack methods on the synthetic dataset in terms of PSNR. PGD (Project Gradient Descent) and C&W (Carlini & Wagner) are white-box attacks. SimBA (Simple Black-box Adversarial Attacks) and SquareA (square attack) are black-box attacks. **Bold** and underline indicate the most severe and the runner-up degradation, respectively. This convention is applied across other tables as well.

Method		DISCNet [3]	UDCUNet [5]	BNUDC [4]	SRUDC [8]	DWFormer [7]	DAGF [27]	UFormer [58]
Clean		35.237	27.427	24.911	21.904	17.651	24.972	19.795
Attack	PGD	$30.166^{↓\;\;5.071}$	$22.936^{↓\;\;4.491}$	14.565↓10.346	$20.656^{↓\;\;1.248}$	$16.484^{↓\;\;1.167}$	$13.772^{↓\;11.200}$	$17.222^{↓\;2.573}$
	C&W	$15.158^{↓\;20.079}$	$14.279^{↓\;13.148}$	7.501↓17.410	$5.985^{↓\;15.919}$	$13.760^{↓\;\;3.891}$	$11.796^{↓\;13.176}$	$17.752^{↓\;2.043}$
	SimBA	$20.578^{↓\;14.659}$	$19.766^{↓\;\;7.661}$	$24.059^{↓\;\;0.852}$	$20.747^{↓\;\;1.157}$	$15.786^{↓\;\;1.865}$	14.794↓10.178	$16.531^{↓\;3.264}$
	SquareA	$25.018^{↓\;10.219}$	$21.557^{↓\;\;5.870}$	$22.986^{↓\;\;1.925}$	$20.300^{↓\;\;1.604}$	$5.985^{↓\;11.666}$	13.454↓11.518	$17.709^{↓\;2.086}$

**Table 2:** Comparison of different attack methods on the synthetic dataset in terms of SSIM. SquareA denotes the square attack [22]. **Bold** and underline indicate the most severe and second most severe degradation, respectively.

Method		DISCNet [3]	UDCUNet [5]	BNUDC [4]	SRUDC [8]	DWFormer [7]	DAGF [27]	UFormer [58]
Clean		0.960	0.937	0.8917	0.834	0.715	0.855	0.642
Attack	PGD	$0.907^{↓\;0.053}$	$0.660^{↓\;0.277}$	0.551↓0.341	$0.648^{↓\;0.186}$	$0.544^{↓\;0.171}$	$0.454^{↓\;0.401}$	$0.543^{↓\;0.099}$
	C&W	0.207↓0.753	$0.237^{↓\;0.7}$	$0.185^{↓\;0.707}$	$0.019^{↓\;0.815}$	$0.157^{↓\;0.558}$	$0.320^{↓\;0.535}$	$0.441^{↓\;0.201}$
	SimBA	$0.529^{↓\;0.431}$	$0.548^{↓\;0.389}$	$0.805^{↓\;0.087}$	$0.715^{↓\;0.119}$	$0.687^{↓\;0.028}$	0.456↓0.399	$0.581^{↓\;0.061}$
	SquareA	$0.559^{↓\;0.401}$	$0.466^{↓\;0.471}$	$0.652^{↓\;0.240}$	$0.469^{↓\;0.365}$	$0.019^{↓\;0.696}$	0.256↓0.599	$0.546^{↓\;0.096}$

**Table 3:** Comparison of different attack objectives in PGD on the synthetic dataset in terms of PSNR. Clean, MSE, and LPIPS denote corresponding clean images, MSE, and LPIPS objectives.

Method	Clean	MSE	LPIPS
DISCNet [3]	35.237	$30.166^{↓\;\;5.071}$	29.870↓5.367
UDCUNet [5]	27.427	$22.936^{↓\;\;4.491}$	$24.293^{↓\;\;3.134}$
BNUDC [4]	24.911	14.565↓10.346	$22.356^{↓\;\;2.555}$
SRUDC [8]	21.904	$20.656^{↓\;\;1.248}$	$19.853^{↓\;\;2.051}$
DWFormer [7]	17.651	$16.484^{↓\;\;1.167}$	$16.658^{↓\;\;0.993}$
DAGF [27]	24.972	$13.772^{↓\;11.200}$	$14.695^{↓\;10.277}$
UFormer [58]	18.795	$17.222^{↓\;\;1.573}$	$17.383^{↓\;\;1.412}$

**Table 4:** Comparison of different attack objectives in PGD on the synthetic dataset in terms of SSIM. Clean, MSE, and LPIPS denote corresponding clean images, MSE, and LPIPS objectives. **Bold** and underline indicate the most severe and second most severe degradation, respectively.

Method	Clean	MSE	LPIPS
DISCNet [3]	0.960	$0.907^{↓\;0.053}$	$0.765^{↓\;0.195}$
UDCUNet [5]	0.937	$0.660^{↓\;0.277}$	$0.694^{↓\;0.243}$
BNUDC [4]	0.892	0.551↓0.341	$0.723^{↓\;0.169}$
SRUDC [8]	0.834	$0.648^{↓\;0.186}$	0.516↓0.318
DWFormer [7]	0.715	$0.544^{↓\;0.171}$	$0.615^{↓\;0.100}$
DAGF [27]	0.855	$0.454^{↓\;0.401}$	$0.484^{↓\;0.371}$
UFormer [58]	0.642	$0.543^{↓\;0.099}$	$0.560^{↓\;0.082}$

**Table 5:** Comparison of adversarial training and our proposed defense strategy in terms of different attack objectives in PGD on the synthetic dataset in terms of PSNR. DP refers to the utilization of DiffPure, whereas FT indicates the implementation of Fine-tuning.

Method	Clean	MSE	LPIPS
DISCNet Adv. [3]	34.082	$30.181^{↓\;3.901}$	$29.379^{↓\;4.703}$
DISCNet [3] + DP	32.773	$31.253^{↓\;1.520}$	$31.286^{↓\;1.487}$
DISCNet [3] + DP + FT	**34.942**	32.167↓2.775	32.493↓2.449
UFormer Adv. [58]	18.819	$18.187^{↓\;0.632}$	$18.205^{↓\;0.614}$
UFormer [58] + DP	18.950	18.391↓0.559	18.766↓0.184
UFormer [58] + DP + FT	**19.938**	$19.312^{↓\;0.626}$	$19.710^{↓\;0.228}$

## Data Availability

The original contributions presented in this study are included in the article. Further inquiries can be directed to the corresponding author.
